# High-Temperature Performance of Polymer-Modified Asphalt Mixes: Preliminary Evaluation of the Usefulness of Standard Technical Index in Polymer-Modified Asphalt

**DOI:** 10.3390/polym11091404

**Published:** 2019-08-27

**Authors:** Kezhen Yan, Lingyun You, Daocheng Wang

**Affiliations:** 1 School of Civil Engineering, Yango University, Fuzhou 350015, China; 2 College of Civil Engineering, Hunan University, Changsha 410082, China; 3 Department of Civil and Environmental Engineering, Michigan Technological University, Houghton, MI 49931, USA; 4 Western Investment Co., Ltd. of CCTEB, Chengdu 610000, China

**Keywords:** polymer-modified asphalt, high-temperature performance, multiple stress creep recovery (MSCR), zero-shear viscosity (ZSV), rutting resistance, correlation

## Abstract

The objectives of this study are to evaluate the high-temperature performance of polymer-modified asphalt and asphalt mixtures, and to investigate if the standard technical indexes are useful in the performance evaluation of the polymer-modified asphalt. There are four typically used polymer-modified asphalt types employed in the study. The standard high-temperature rheological test, such as the temperature sweep test, was used to express the high-temperature performance of the polymer-modified asphalt. Also, considering the non-Newtonian fluid properties of the polymer-modified asphalt, the multiple stress creep recovery (MSCR) and zero-shear viscosity (ZSV) tests were employed for the characterizations. Besides, based on the mixture design of SMA-13, the high temperature of the polymer-modified asphalt mixture was evaluated via Marshall stability and rutting tests. The test results concluded that the ranking of the four kinds of polymer-modified asphalt was different in various laboratory tests. The TB-APAO has the best technical indexes in MSCR and ZSV tests, while the WTR-APAO performed best in the temperature sweep test. In addition, the correlation between the polymer-modified asphalt and the asphalt mixture was very poor. Thus, the present standard technical indexes for the profoundly polymer-modified asphalt mixtures are no longer suitable.

## 1. Introduction

The main research result of the SHRP program initiated in the 1980s was the Superpave asphalt performance evaluation system. In this evaluation system, the complex shear modulus G*, phase angle δ, and rutting indicator G*/sinδ obtained from the dynamic shear rheological test are the leading technical indexes for evaluating the mechanical response of asphalt at high-temperatures [1,2,3,4]. Also, considering that the asphalt is a kind of viscoelastic material, typically, G_e_ = G*cosδ and G_v_ = G*sinδ are defined as the elastic and viscous portions of the material, respectively. In the evaluation of the low-temperature properties of asphalt, the bending beam rheometer test is typically used for research. According to the SHRP research results, the stiffness modulus *S* and the stiffness dissipation rate *m*-value are the primary basis for characterizing the low-temperature performance of asphalt [2,3,5]. With the modified asphalt in the field and the extensive use of pavement engineering, the properties of the modified asphalt have changed to no small extent from the original asphalt. Thus, it remains to be understood whether the current standard technical indexes are useful in the modified asphalt.

Nowadays, since the polymer is widely used as an asphalt modifier, the material properties and physical and chemical properties of the modified asphalt will change to a large extent. For example, polymers that have been used to modify asphalt include styrene-butadiene-styrene (SBS), styrene-butadiene rubber (SBR), Elvaloy^®^, rubber, ethylene-vinyl acetate (EVA), polyethylene, and others [6,7]. Desirable values of polymer-modified asphalts include more excellent elastic recovery, a higher softening point, greater viscosity, greater cohesive strength, and more excellent ductility [8]. Also, Yan et al. [9,10,11] reported that the present polymer modified asphalt should be further modified, such as the anti-aging agent of amorphous poly alpha olefin (APAO), for improving the compressive performances. Besides, several studies proposed the profoundly rubberized asphalt with the rubber particles content of around 20 wt %, a promising technology in producing crumb rubber asphalt as it overcomes the shortcomings such as lack of storage stability and workability [12,13,14]. Lin et al. [15] studied the performance characteristics of terminal blend (TB) rubberized asphalt with SBS and polyphosphoric acid. The physicochemical properties of the polymer-modified asphalt have been significantly changed compared with the non-modified asphalt. Therefore, whether the Superpave system is applicable to the pavement construction materials, in the last century, which reflects the actual performance of the polymer-modified asphalt, is worthy of further study. To this end, since the beginning of this century, many scholars and scientific research institutions have done much research to explore new performance evaluation indexes of modified asphalt.

In the works in 1993, Sybilski [16] believed that polymer modified asphalt differs from ordinary viscoelastic materials in its material properties and that its chemical and physical properties are different from those of matrix asphalt. If the matrix asphalt is still used to judge the polymerization, modified asphalt will cause significant errors. Since the polymer modified asphalt is mainly present in the form of non-Newtonian fluid at high temperatures, the viscosity test results will vary with the shear rate. In order to eliminate the influence of shear rate on the test results during measurement, Sybilski [17] proposed to introduce the concept of zero-shear viscosity (ZSV) in non-Newtonian fluid theory into polymer modified asphalt to judge asphalt for high-temperature performances. 

Also, except for the ZSV test, the AASHTO and ASTM specifications incorporated the multiple stress creep recovery (MSCR) test into the polymer-modified asphalt as the high-temperature performance evaluation method [18,19]. According to the AASHTO TP70 test procedure [20], 0.1 and 3.2 kPa were used as test loads, and the control stress model was used to conduct performance tests under two different load levels. The irreversible deformation compliance Jnr, deformation recovery rate, and stress response difference index were used to evaluate the high-temperature performance of asphalt. The MSCR test method has been recognized by many scientific research institutions in the United States. Relevant research results show that the MSCR test method is useful for predicting the high-temperature stability of asphalt [21,22,23]. Mehta and DuBois et al. [24,25] indicated through experimental research that in the polymer modified asphalt, asphalt cement performance index, and the high-temperature stability of the mixture have a strong correlation, and the correlation between the rutting factor and the high-temperature performance of asphalt is weak. Because the dynamic response of polymer-modified asphalt under large strain load is nonlinear, if the test load is too small, it cannot reflect the resistance of the material to permanent deformation [26,27]. Through experimental research, Dreessen et al. [28] believe that the MSCR test results under higher stress load have a better correlation with the high-temperature stability of the asphalt mixture. Golalipour et al. [29] analyzed the test results and the actual load and consider that the existing test load is smaller than the actual road load. It is better to adjust the load to 10 kPa. Therefore, in this study, the ZSV and MSCR (with a load of 3.2 kPa/10 kPa) were used for the high-temperature performance evaluations with the standard technical indexes from the rheological tests. 

Furthermore, the correlation between the performance of the polymer-modified asphalt mixture and the polymer-modified asphalt is a vital issue. For example, the rutting resistance of the asphalt mixture is an essential technical index for characterizing the high-temperature performances in pavement engineering [30,31,32,33,34,35,36]. Therefore, the objectives of this study are to evaluate the high-temperature performance of the selected kinds of polymer-modified asphalt via standard DSR test and the newly developed ZSV and MSCR test and to investigate the corresponding high-temperature performance of the asphalt mixtures. The corresponding high-temperature performance of the asphalt mixtures was characterized via Marshall test and rutting test. Also, the correlations between the different technical indexes were analyzed. 

## 2. Materials and Asphalt Mixture Design

Because the standard technical indexes are useful in the regular asphalt and asphalt mixture [37,38,39], the study is to characterize the possibility of applying the standard technical indexes in the polymer-modified asphalt. Thus, in this study, there are four typically used polymer-modified asphalt employed, which include the SBS (styrene-butadiene-styrene) modified asphalt, EVA (ethyl vinyl acetate) modified asphalt, WTR (waste tire rubber) and APAO (amorphous-poly-alpha-olefin) composite modified asphalt, and APAO modified TB (terminal blend) asphalt. The TB asphalt is the profoundly modified rubberized asphalt. Note that, since the SBS is a high price modifier, one of the objectives of the study is to evaluate if it is possible to find possible options for replacing the SBS modified asphalt. Thus, based on the literature review [40,41,42], the rubberized asphalt, i.e., WTR and TB asphalt, were further modified via APAO, in order to further improve their performance. 

Here, the original asphalt used for preparing SBS, EVA, and WTR-APAO modified asphalt was AH-90A, from Yueyang Jinling Petrochemical (Yueyang, China), while TB asphalt with the dosage of 20% rubber particles (wt.) was from the key laboratory of road and traffic engineering, Tongji University (Shanghai, China). Note that, in this study, SBS modified asphalt is a finished modified asphalt with around 4.5 wt % SBS, from Yueyang Jinling Petrochemical (Yueyang, China), while the other three types of modified asphalt were produced in the laboratory. The modifying agent of EVA used was an Evatane^®^ 4269 produced by DuPont (Wilmington, DE, USA), with a vinyl acetate dosage of 28 wt % and a melting point of 72 °C. The APAO used in the modification had a density of 1.41 g/cm^3^ and a softening point of 295 °C. Also, the WTR employed in the modification was derived from the ambient grind and the mean particle size of the WTR particles, determined by screening, was 0.29 mm. The main technical indexes of these four types of asphalt are demonstrated in Table 1, which meet the standard requirements [43].

The EVA modified asphalt samples were prepared using a high shear laboratory type mixer rotating at 3000 rpm for 30 min. In preparation, the original AH-90A asphalt was heated to fluid condition (170 °C). After 30 min high-speed shearing processes, in order to ensure the modifying agent and the asphalt were sufficiently cross-linked, the mixtures were curing at the same temperature for 20 min. The concentrations of EVA in AH-90A were chosen a 6 wt %, and the EVA modified asphalt is referred to as EVA in this study. Meanwhile, the WTR and APAO composite modified asphalts (refers to WTR-APAO in this study) were prepared by using the same procedures as mentioned above. The concentrations of WTR and APAO particles in the asphalt were 15 and 4 wt %, respectively. Furthermore, the APAO modified TB asphalt (which refers to TB-APAO in this study) with the dosage of APAO particles of 6 wt %was created by using the same mixing and curing procedures of EVA. 

Also, in this study, the stone mastic asphalt (SMA-13) mixtures with the four kinds of asphalt were used for the high-temperature performance evaluations. Table 2 shows the detailed gradation of SMA-13. Note that the intermediate gradation in Table 2 was used in SMA-13 mixture preparations. Compared with the ordinary asphalt mixture, SMA has a tremendous amount of coarse aggregate, mineral powder, and asphalt, while there are few usages in fine aggregates. The aggregate used was basalt stone with the average bulk gravity (dry) of 2.895, and the employed mineral powder is limestone with the average bulk gravity (dry) of 2.701. Besides, because the amount of asphalt and mineral powder in SMA-13 is more considerable than that of the conventional asphalt mixture, in order to ensure the stable performance of the mixture after mixing, an appropriate amount of fiber stabilizer generally needs to be added to the mixture during the mixing. Here, a road using wood fibers (lignocellulosic biomass), with an average length of 1.1 mm, and an oil absorption rate of 6.83 times fiber quality, was used in the SMA mixture preparations. 

It is estimated that 6.0 wt % is the optimum asphalt content (OAC). Five asphalt contents 5.4, 5.7, 6.0, 6.3, and 6.6 wt %were selected as the possible OAC for applying in the Marshall test to determine the OAC of SMA-13 mixtures used. Based on the Chinese standard of JTG f40-2004 [44], the test data of bulk specific gravity (*Gmb*), air void (*VV*), voids in mineral aggregate (*VMA*), voids filled with asphalt (*VFA*), Marshall Stability (*MS*), and flow value (*FL*) were demonstrated in Table 3. The determined OAC in the study was 6.11 wt %, and the *VV* and *VMA* were 3.741% and 17.052%, respectively. Note that the Marshall tests mentioned earlier were based on the SBS asphalt binder. The different asphalt binders have various OACs; in order to control the variables in the study, all of the SMA-13 mixtures used the same asphalt content, i.e., OAC of SBS asphalt binder.

## 3. Testing Methods

The Dynamic Shear Rheometer (DSR) was applied for investigating the high-temperature rheological properties through temperature sweep, multiple stress creeps recovery (MSCR), and zero-shear viscosity (ZSV) tests. The temperature sweep tests were conducted at the fixed frequency of 10 rad/s according to AASHTO T315 [45]. The test temperature ranged from 30–90 °C with the temperature step of 2 °C/min. The employed sample diameter and thickness were 25 and 1 mm, respectively. The rheological properties, such as complex modulus (G*), phase angle (δ), and rutting indicator (G*/sin(δ)), were used to express the high-temperature rheological properties of the proposed asphalt from the temperature sweep tests. Note that three replicates were used for each type of asphalt.

Also, the MSCR tests were conducted via DSR to apply a continuous cyclic load to the asphalt to determine the degree of creep deformation and the deformation recovery. The MSCR test was used on the rolling thin film oven (RTFO) residue of asphalt, which is applied to simulate the short-term aging condition of asphalt. Note that the RTFO aging processes with a temperature of 163 °C for 85 min [46]. Besides, the MSCR sample diameter and thickness were 25 and 1 mm, respectively. According to AASHTO TP70-12 [20], the MSCR test was conducted at 60 °C. Each cycle consisted of 1 s shear creep followed by a recovery period of 9 s [47]. The first 10 cycles of creep and recovery were conducted under the shear load of 3.2 kPa, and then a 10 kPa of the shear load was employed on the sample for another 10 cycles. Note that, during the MSCR test, three replicates were used for each type of asphalt.

Besides, Sybilski [16,17] believes that for pseudo plastic fluids such as asphalt, the results of the viscoelasticity test are closely related to the shear rate, and the result that best reflects the material properties is the ideal shear rate at zero. Therefore, three kinds of ZSV tests were employed to study the polymer-modified asphalts, such as the Cross/Sybilski model, Cross/Williamson model, and Carreau model. For the ZSV test, the frequency ranged from 100 to 0.1 rad/s with the test temperature of 60 °C. The ZSV sample diameter and thickness were 25 and 1 mm, respectively. Note that, during the ZSV test, three replicates were used for each type of asphalt. The simplified equation for the Cross/Sybilski model as shown in Equation (1), where $\eta^{*}$ is the complex viscosity with the unit of Pa·s, $\eta_{0}^{*}$ is the zero-shear viscosity (ZSV) with the unit of Pa*s, ω is the frequency with the unit of rad/s, *K* and *m* are the coefficients related to the physicochemical properties of asphalt. 

(1) $$\eta^{*}=\frac{\eta_{0}^{*}}{1+{\left(K\omega \right)}^{m}}$$

The Cross/Sybilski model is obtained by ignoring the viscosity of the asphalt when the frequency is infinite, and the polymer modified asphalt has multiple properties. For some high-viscosity-elastic materials, such as polymer modified asphalt, even if the frequency is significant, the viscosity is still not negligible. Therefore, it is necessary to use the Cross/ Williamson model for data analysis of ZSV. The Cross/ Williamson model can be expressed as follows:

(2) $$\eta^{*}=\frac{\eta_{0}^{*}-\eta_{\infty }^{*}}{1+{\left(K\omega \right)}^{m}}+\eta_{\infty }^{*}$$

where $\eta_{\infty }^{*}$ is the complex viscosity of asphalt when the shear frequency is infinite, here, $\eta_{\infty }^{*}$ was defined as the complex viscosity at 100 rad/s since the actual $\eta_{\infty }^{*}$ cannot be achieved from the laboratory tests. Except for the above-mentioned two model, the Carreau model is also usually used for the analysis of ZSV. The Carreau model is similar in form to the Cross model. The biggest difference between these two is that the former will quickly form a gentle curve in the low-frequency domain. Therefore, the ZSV values obtained by the Carreau model are usually relatively small. The expression of the Carreau model is illustrated in Equation (3).

(3) $$\eta^{*}=\frac{\eta_{0}^{*}-\eta_{\infty }^{*}}{{\left[1+{\left(K\omega \right)}^{2}\right]}^{m/2}}+\eta_{\infty }^{*}$$

In addition, the Marshall stability test and the rutting test were employed for evaluating the high-temperature performance of the corresponding asphalt mixtures. The test step of Marshall stability was carried out following the Chinese standard of JTG E20-2011 [43]; before conducting the Marshall stability test, the samples were cured in a 60 °C constant temperature water bath for 30 min. Also, the test temperature was 60 °C. During the Marshall stability test, the recorded maximum load before the failure of the Marshall samples is the Marshall stability (*MS*) with the unit of kN. Meanwhile, the corresponding vertical deformation is the flow value (*FL*) with the unit of mm. The Marshall modulus (*T*) can be calculated via Equation (4). Note that, during the Marshall stability test, there are at least three replicates used for each type of asphalt mixture.

(4) $$T=\frac{MS}{FL}$$

Furthermore, according to the Chinese standard of JTG E20-2011[43], for the rutting test, the asphalt mixture samples should be prepared with the size of 300 × 300 × 50 (*L* × *b* × *h*) mm^3^. Before applying rutting tests, the asphalt mixture samples were cured in a 60 °C constant temperature bath for 5–12 h. The test temperature was 60 °C. The contact pressure between the wheels and the samples was 0.7 MPa, and the distance for a single passing was 23 cm. The dynamic stability (*DS*) of the samples can be calculated via Equation (5), where *d*_1_ and *d*_2_ are the rutting depth at *t*_1_ (45 min) and *t*_2_ (60 min), respectively, and *N* is the speed of the wheel passing over the center of the sample (42 cycles/min). Note that, during the rutting test, there are at least three replicates used for each type of asphalt mixture.

(5) $$DS=\frac{\left(t_{2}-t_{1}\right)}{\left(d_{2}-d_{1}\right)}*N$$

## 4. Laboratory Results and Analysis

### 4.1. High-temperature Rheological Properties of Asphalt

**Figure 1:**
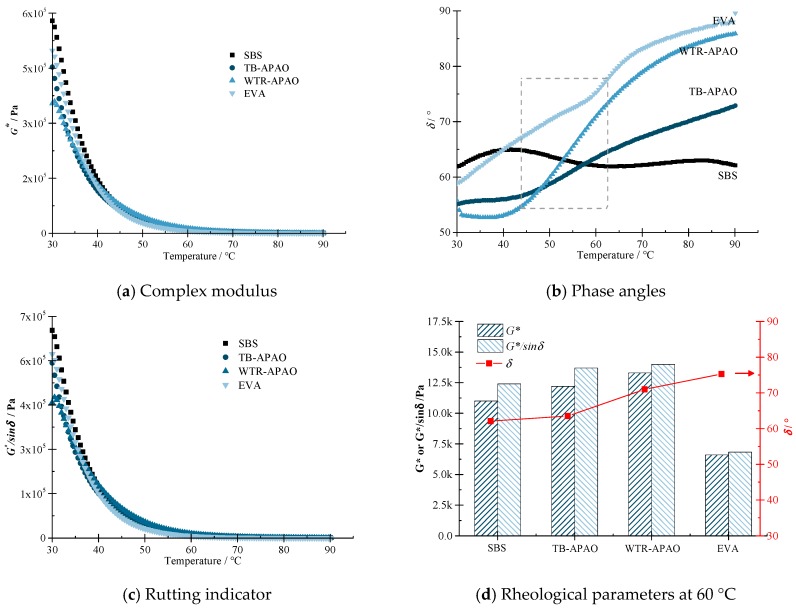
High-temperature rheological properties of asphalts via temperature sweep tests: (**a**) complex modulus, (**b**) phase angles, (**c**) rutting indicator, and (**d**) rheological parameters at 60 °C.

Temperature sweep test results. The rheological properties of the four kinds of polymer modified asphalt are shown in Figure 1, where the complex modulus G*, phase angle δ, and rutting indicator G*/sin δ are depicted in Figure 1a–c, respectively. Observing Figure 1b, it is not difficult to find that the phase angle curves have different forms. In the temperature range of 40–60 °C, the four curves have the most extensive change range, which reflects the temperature of various modifiers in this temperature range. The difference in response to change is most pronounced. Among them, SBS has similarities with TB-APAO, the trend of rising of both curves has slowed down, and there has been a plateau or even a certain degree of decline, while the phase angle of the other two modified asphalts was still rising. The phase angle is an essential characterization of the viscosity change of asphalt under the action of external shear force, which reflects that SBS and TB-APAO can still maintain relatively good shear strain recovery ability at higher temperatures. The SBS modified asphalt was preferred.

Besides, it is found, from Figure 1a,c, that in the interval of 30–40 °C, the complex modulus G* and the rutting indicator G*/sin δ value of SBS was significantly larger than the other three kinds of asphalt. When the temperature is greater than 60 °C, the rutting indicator G*/sin δ results of WTR-APAO was in the first place, while the high-temperature performance of EVA modified asphalt was always weak. 

The study selected the performance of the modified asphalt at 60 °C for the detailed evaluations, here, Figure 1d summarizes the rheological properties of the four modified asphalts at 60 °C. It can be observed that the proposed EVA presented the lowest rutting indicator G*/sin δ and complex modulus G*, while depicting the highest phase angle δ. Thus, the high-temperature performance of EVA performed most poorly, while those of WTR-APAO performed best, followed by TB-APAO and SBS. Note that the difference between the three modified asphalts, i.e., WTR-APAO, TB-APAO, and SBS, was less than 20%, while the difference between WTR-APAO and EVA was higher than 60%. Therefore, from the temperature sweep test, it is concluded that, except for EVA, the proposed WTR-APAO and TB-APAO can be considered for replacing the traditional SBS.

However, based on the performance-graded (PG) asphalt binder specification (from AASHTO MP1 [48]), the high-temperature grades of the polymer-modified asphalt were 76, 82, 76, and 64 °C, for SBS, TB-APAO, WTR-APAO, and EVA, respectively. 

**Figure 2:**
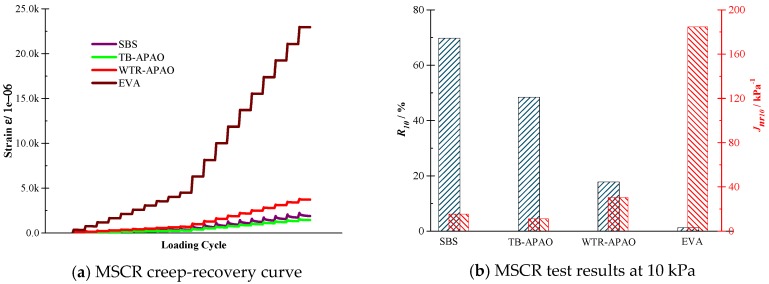
Multiple stress creep recovery (MSCR) test results of the polymer-modified asphalt at 60 °C: (**a**) MSCR creep–recovery curve and (**b**) MSCR test results at 10 kPa.

MSCR test results. Figure 2 displays the MSCR test results of the polymer-modified asphalt at 60 °C. In Figure 2a each loading cycle includes a one-second creep phase and a nine-second recovery phase. There are multiple loading cycles presented for the four polymer-modified asphalts. In the creep phase, the actual strain level went up with the increase of the loading time. In the recovery phase section, the strain recovered at the beginning but the recovery rate reduced with the loading time. The results expressed the significant visco-elastic-plastic properties of the polymer-modified asphalt. Figure 2b illustrates the creep and recovery rate of the asphalt at 10 kPa. Under the creep load condition, the EVA has a significant degree of deformation and a low deformation recovery rate, which indicated that the EVA performed soft-viscosities compared with the other asphalt. However, the TB-APAO and SBS performed best, followed by the WTR-APAO because these modified asphalts have the least deformation and the most substantial deformation recovery ability in the MSCR test. 

**Figure 3:**
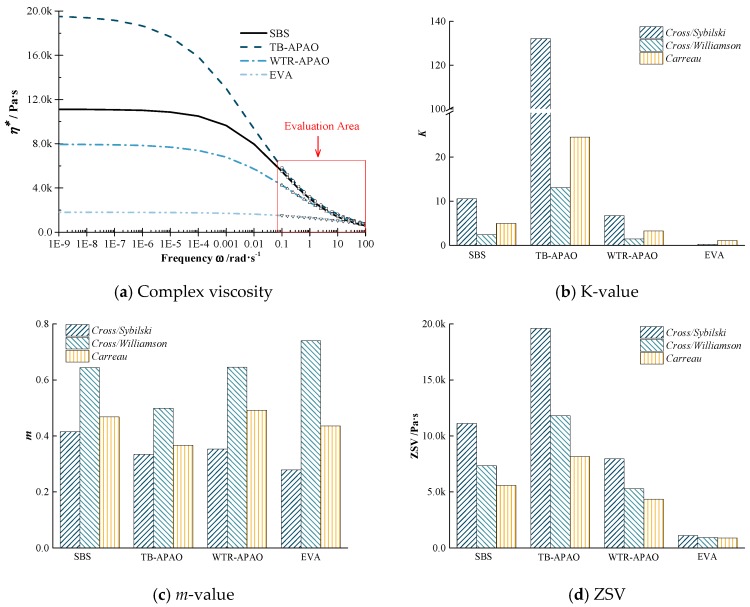
Zero-shear viscosity (ZSV) test results of modified asphalt at 60 °C: (**a**) complex viscosity, (**b**) *K*-value, (**c**) *m*-value, and (**d**) ZSV.

ZSV test results. The ZSV test results of the modified asphalt at 60 °C are depicted in Figure 3, which include the complex viscosity–frequency curves, the fitted *K*- and *m*-value, and the fitted ZSV-value using three different kinds of ZSV models. 

From Figure 3, it can be seen that there are substantial differences in *K-* and *m-*value between different types of modified asphalt. However, what they have in common is that the *m*-value is always small, and the *K*-value fluctuates greatly between different modified asphalts. For the overall fitting situations, the larger the ZSV-fitting result, the larger the *K*-value. According to Sybilski’s note in the reference [16], the parameter *m* mainly reflects the sensitivity of the asphalt to shear stress, and the closer the *K-* and *m*-value is to zero, the closer the performance of the material is to the Newtonian fluid. 

Also, from Figure 3b, among the *K*-values obtained based on the Cross/Sybilski model, TB-APAO was the highest, which reflects that the profoundly modified rubber asphalt was very similar to the non-Newtonian fluid. Besides, from Figure 3c, it can be observed that the *m*-values of the four kinds of modified asphalt were calculated to be above 0.3, indicating that these four kinds of modified asphalts were sensitive to shearing, and the viscosity values obtained by shearing at different frequencies will be different, among which the Cross/William model is calculated. The EVA has the most considerable *m*-value, indicating that the viscosity test result is most affected by the shear frequency. Figure 3d illustrated that the ZSV fitting results obtained by the three calculation models are different. Among them, the zero shear viscosity value obtained by the Cross/Sybilski model was the largest, and the difference between different types of asphalt was relatively significant. Thus, the test confirmed that the profoundly modified asphalt has been similar to the non-Newtonian fluid, and it is necessary to analyze the high-temperature performance with zero shear viscosity. In order to distinguish the high-temperature performance of different asphalts by using zero shear viscosity, it is necessary to select the calculation results obtained by the differentiated Cross/Sybilski model.

In addition, from Figure 3d, it can be concluded that the proposed TB-APAO performed best to resist high-temperature deformations, followed by the SBS, WTR-APAO, and EVA. Note that the ZSV of EVA is significantly less than the other three polymer-modified asphalts, which indicated that, like the MSCR and temperature sweep test results, the EVA performance was significantly soft-viscoelastic. Thus, except for EVA, as expected, the WTR-APAO and TB-APAO modified asphalt can be considered for replacing the conventional SBS modified asphalt. 

In summary, according to the above-mentioned the temperature sweep test, MSCR test, and ZSV test for the polymer-modified asphalt, the ranking for the high-temperature performance of the proposed four kinds of polymer-modified asphalt was different for the different test methods. The MSCR test and ZSV test results concluded that the TB-APAO performed best, followed by the SBS, WTR-APAO, and EVA, while the temperature sweep test results concluded that the WTR-APAO performed best, followed by the TB-APAO, SBS, and EVA. 

### 4.2. High-Temperature Performance of Asphalt Mixtures

In the pavement construction field, asphalt is often used as a form of asphalt pavements, and the performance of the asphalt mixture usually characterizes its real performance. Therefore, in order to characterize and rank the actual performance of polymer-modified asphalt in pavement constructions, it is indeed necessary to evaluate the corresponding performance of the asphalt mixtures. The laboratory high-temperature performance of the polymer-modified asphalt mixture was expressed via the Marshall test and rutting test in this study. Table 4 and Table 5 demonstrated the test results of these two tests. 

From Table 4, it can be seen that the TB-APAO asphalt mixture performed the highest Marshall stability (*MS*, 8.9 kN), followed by the WTR-APAO, SBS, and EVA asphalt mixtures. The results concluded that the EVA and SBS asphalt mixture performed more softly than others. The Marshall Modulus (*T*) results proved the aforementioned changing trend. Thus, except for the EVA asphalt mixture, the TB-APAO and WTR-APAO asphalt mixtures have better Marshall stability performance compared with the traditional SBS asphalt mixture under the high-temperature conditions.

From Table 5, it can be seen that the dynamic stability (*DS*) of TB-APAO asphalt mixture was 11,340 times/mm, while the *DS* of SBS, WTR-APAO, and EVA asphalt mixture were 7787, 5989, and 2655 times/mm, respectively. The *DS* results concluded that the proposed TB-APAO modified asphalt mixture performed best for resisting the rutting deformations. However, the parameters of *D*45 and *D*60, i.e., the rutting depth of the samples at 45 and 60 min, respectively, showed the different ranking information for these four asphalt mixtures. The EVA asphalt mixture performed best at 45 and 60 min to resist deformation, while its *DS* results were the worst compared with the others. Also, it can be seen that the ranking information from the Marshall Stability test and the rutting test was different.

### 4.3. Comprehensive Analysis for the Technical Indexes

In this section, in order to comprehensively evaluate the high-temperature performance of the polymer-modified asphalt and the corresponded asphalt mixtures, the technical indexes were normalized in a range from 0 to 100. The maximum test value of a high-temperature performance index of four kinds of asphalt was the standard series. The test values of different high-temperature technical indexes of asphalt were comparative series. The technical indexes were normalized via Equation (6), where *x* is the technical index, such as softening point, viscosity, rutting indicator, recovery, ZSV, and high-temperature of PG grade of the asphalt, *k* is the kind of polymer modifier, which includes SBS, TB-APAO, WTR-APAO, and EVA. Also, the high-temperature technical indexes of the asphalt mixtures were normalized via the equation.

(6) $$Normalized\;x_{i}\left(k\right)=\frac{x_{i}\left(k\right)}{x_{max}\left(k\right)}*100$$

**Figure 4:**
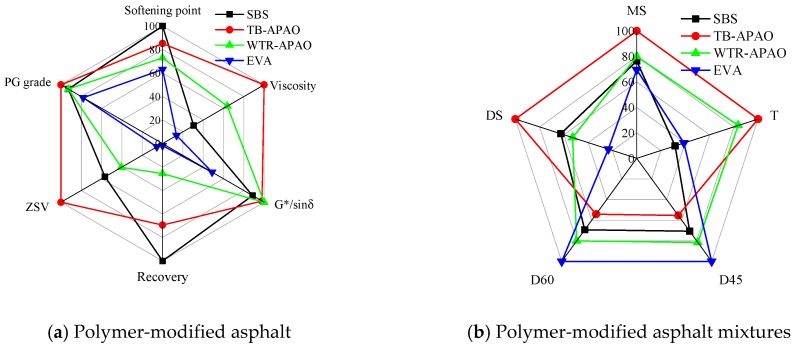
High-temperature performance distributions in hexagonal envelope diagram: (**a**) polymer-modified asphalt and (**b**) polymer-modified asphalt mixtures.

Figure 4 represented the normalized technical indexes of the asphalt and asphalt mixtures. From Figure 4a, the asphalt material with the largest hexagonal envelope area should have the best high-temperature overall performance. Thus, the comprehensive high-temperature performance ranking of the polymer-modified asphalt was TB-APAO, followed by SBS, WTR-APAO, and EVA. Note that dynamic stability (*DS*) is a useful indicator for characterizing the high-temperature performance of asphalt mixtures [49,50,51,52]. Therefore, in order to create the relationship between the performance of asphalt and asphalt mixtures, the *DS* results were selected for the comparison. Here, by comparing with the normalized results of the asphalt mixtures, depicted in Figure 4b, the ranking information of the polymer-modified asphalt was the same as that of the *DS* of the asphalt mixtures. 

However, the presented comprehensive analysis results were a good match with the MSCR test and ZSV test results, while different from the traditional temperature sweep test results. Besides, although the proposed comprehensive analysis and the MSCR and ZSV test matched the *DS* in the asphalt mixtures, the other technical indexes of the asphalt mixture did not correlate with the performance of the polymer-modified asphalt. Thus, the present standard technical indexes of the asphalt and the asphalt mixtures may no longer be suitable for evaluating the polymer-modified asphalt mixtures. The profoundly polymer-modified asphalt may cause it to be very similar to the non-Newtonian fluid, while the standard technical indexes are typical for the Newtonian fluid un-modified asphaltic materials.

## 5. Summary, Conclusions, and Future Research Works

In order to assess whether the technical indexes for the standard asphalt and asphalt mixtures are suitable for the profoundly polymer-modified asphalt mixtures, this study evaluated the high-temperature performance of the four kinds of polymer-modified asphalt. The standard high-temperature rheological test, such as the temperature sweep test, was used to express the high-temperature performance of the polymer-modified asphalt. Also, considering the non-Newtonian fluid properties of the polymer-modified asphalt, the MSCR and ZSV test were employed for the characterizations. Besides, based on the mixture design of SMA-13, the high-temperature of the polymer-modified asphalt mixture was evaluated via Marshall Stability and rutting tests. According to the laboratory tests, the main conclusions are as follows.

(1) From the high-temperature rheological properties tests, the TB-APAO and WTR-APAO can be considered for replacing the traditional SBS. Because of the technical indexes represented that the TB-APAO and WTR-APAO performed better than SBS. 

(2) The ranking of the four kinds of polymer-modified asphalt from different tests was different. The TB-APAO has the best technical indexes in the MSCR and ZSV tests, while the WTR-APAO performed best in the temperature sweep test. The MSCR and ZSV tests results were a good match with the dynamic stability of the asphalt mixtures. 

(3) According to the preliminary results and the simple discussions on the proposed four kinds of asphalt and its mixtures, it is found that the correlation between the polymer-modified asphalt and the asphalt mixture was very poor. Thus, the present standard technical indexes for the profoundly polymer-modified asphalt mixtures may no longer be suitable. However, the accuracy of the conclusion should be further proved via various laboratory tests for the polymer-modified asphalt and its mixture.

Future research should focus on the development of technical indexes for characterizing the high-temperature performance of the polymer-modified asphalt mixtures. Besides, the present study only was used for the evaluations of the high-temperature performance; the functions of the standard technical indexes for the investigating the low-temperature performance should be proposed in the future. 

In addition, the research on the performance evaluation index of asphalt is inseparable from the performance of the asphalt mixture. Thus, the related mathematical method, such as the grey-correlation analysis, can be used in the correlation analysis between the technical indexes of asphalt and asphalt mixtures.

## Figures and Tables

**Table 1:** Main Technical Index of Used Four Kinds of Asphalt (According to the Chinese standard of JTG E20-2011 [43]).

Items	Unit		Modified Asphalt Binders				Test Standard Code
		AH-90A	SBS	EVA	WTR-APAO	TB-APAO	
Penetration at 25 °C	0.1 mm	92.0	71.1	61.2	46.2	46.4	T0604
Penetration index	n/a	−0.80	0.80	1.09	2.96	1.24	T0604
Ductility at 5 °C	cm	-	44.7	12.6	10.5	34.6	T0605
Softening point	°C	49.1	90.2	56.9	65.7	76.8	T0606
Viscosity at 135 °C	Pa*s	0.52	2.05	0.93	4.28	6.71	T0625

Note that the study only tested the ductility of AH-90A at 10 °C without testing at 5 °C, the ductility of AH-90A at 10 °C is 135.0 cm.

**Table 2:** Gradation of Aggregate Used for SMA-13 (According to the Chinese standard of JTG F40-2004 [44]).

Sieve Size (mm)	16	13.2	9.5	4.75	2.36	1.18	0.6	0.3	0.15	0.075
Upper (%)	100	100	75	34	26	24	20	16	15	12
Lower (%)	100	90	50	20	15	14	12	10	9	8
Intermediate (%)	100	95	62	27	20.5	19	16	13	12	10

**Table 3:** Testing Data for Determining Optimum Asphalt Content (OAC)**.**

Asphalt Content (%)	5.4	5.7	6.0	6.3	6.6
*Gmb*	2.448	2.450	2.454	2.450	2.440
*VV* (%)	4.968	4.461	3.820	3.538	3.479
*VMA* (%)	16.571	16.779	16.880	17.290	17.897
*VFA* (%)	70.020	73.414	77.373	79.540	80.562
*MS* (kN)	7.060	7.530	7.900	6.760	6.240
*FL* (0.1 mm)	44.000	44.800	35.400	33.200	32.350

**Table 4:** Marshall test results of asphalt mixtures.

Mixture Types	Marshall Stability *MS*/ kN	Marshall Modulus *T*/ kN*mm^−1^
SBS	6.80	1.97
TB-APAO	8.90	6.22
WTR-APAO	7.12	5.20
EVA	6.19	2.42

**Table 5:** Rutting test results of asphalt mixtures.

Mixture Types	*D*45/mm	*D*60/mm	*DS*/times·mm^−1^
SBS	4.02	4.11	7087
TB-APAO	3.15	3.21	11,340
WTR-APAO	4.64	4.75	5989
EVA	5.69	5.93	2655
